# Experiences of pre-hospital emergency medical personnel in ethical decision-making: a qualitative study

**DOI:** 10.1186/s12910-018-0334-x

**Published:** 2018-12-19

**Authors:** Mohammad Torabi, Fariba Borhani, Abbas Abbaszadeh, Foroozan Atashzadeh-Shoorideh

**Affiliations:** 1 Department of Medical Surgical Nursing, School of Nahavand Paramedical, Hamadan University of Medical Sciences, Hamadan, Iran; 2 Medical Ethics and Law Research Center, Shahid Beheshti University of Medical Sciences, Tehran, Iran; 3 Department of Medical Surgical Nursing, School of Nursing & Midwifery, Shahid Beheshti University of Medical Sciences, Tehran, Iran; 4 Bam University of Medical Sciences, Bam, Iran; 5 Department of Psychiatric Nursing, School of Nursing & Midwifery, Shahid Beheshti University of Medical Sciences, Tehran, Iran; 6 Department of Nursing, School of Nursing & Midwifery, Shahid Beheshti University of Medical Sciences, Vali-Asr Avenue, Cross of Vali-Asr and Neiaiesh Highway, Opposite to Rajaee Heart Hospital, Tehran, 1996835119 Iran

**Keywords:** Ethical decision-making, Emergency medical services, Content analysis, Pre-hospital, Qualitative research

## Abstract

**Background:**

Emergency care providers regularly deal with ethical dilemmas that must be addressed. In comparison with in-hospital nurses, emergency medical service (EMS) personnel are faced with more problems such as distance to resources including personnel, medico-technical aids, and information; the unpredictable atmosphere at the scene; arriving at the crime scene and providing emergency care for accident victims and patients at home. As a result of stressfulness, unpredictability, and often the life threatening nature of tasks that ambulance professionals have to deal with every day, ethical decision-making (EDM) has become an inevitable challenge.

**Methods:**

The content analysis approach was used to conduct the present qualitative study in Iran. The participants consisted of 14 EMS personnel selected through purposive sampling, which continued until the data became saturated. Data were collected using semi-structured interviews and analyzed concurrently with their collection through the constant comparison method.

**Results:**

The process of data analysis resulted in the emergence of 3 main categories “respecting client’s values”, “performing tasks within the professional manner”, “personal characteristic”, and the emergence of eight (8) sub-categories signifying participants’ experiences with regard to EDM.

**Conclusion:**

According to the results, when EMS personnel are faced with ethical dilemmas, they consider the client’s values and professional dignity, and perform the assigned tasks within the framework of the regulation. The findings also suggest that pre-hospital care providers assess legal consequences before making any decision. Further studies should be conducted regarding the experiences of the subordinates and other related parties.

## Background

Emergency Medical Services (EMS) are the first level of health care provided in out-of-hospital medical emergency departments [1]. Medical emergency system is a part of the chain system in the care of patients and it continues from the time of the event to the patient’s rehabilitation and discharge [2]. Its purpose is to provide care, ensure immediate transfer of patients, and reduce mortality [3]. In dangerous and unpredictable care situations, ambulance professionals are often faced with situations in which decision-making puts them under heavy pressure, especially when they are faced with ethical challenges [4]. When faced with ethical challenges, the EMS personnel should be able to make an ethical decision at a minimum time in emergencies [5]. In comparison with other paramedical professionals, the decision-making conditions for the EMS personnel is different in terms of the medical care field. It includes distance to resources such as personnel, medico-technical aids and information; the necessity to perform cardiopulmonary resuscitation (CPR) at the scene; facing the crime scene; family expectations, the requests of client’s relatives and working tightly within a small team [5, 6]. Some of the ethical challenges which pre-hospital emergency caregivers (PECs) face include: intervening in dangerous situations, protecting themselves and the ambulance [7], obtaining informed consent [8] and determining the decision-making capacity, [9] refusal of treatment and transport [10], transferring non-emergency patients [11], misplaced requests [12], respecting the patient’s privacy [13, 14], withholding CPR, and futile resuscitation [15].

In Iran, EMS staff consists of nurses, emergency medical technicians (EMTs), emergency medical experts, and other relevant academic disciplines (operating room and anesthesia technicians). In addition, a pre-hospital emergency medical school has been established in less than a decade in Iran. Therefore, most of the human resources employed in EMS are nurses. Personnel with different grades and disciplines, such as nursing and medical emergencies, are all within the scope of this study and are considered as a professional collection of out-of-hospital emergency medical services. The duties of each EMS staff are clearly defined and they act in decision-making based on guidelines and available resources.

The results of Ebrahimian et al. qualitative research on the experiences of EMS personnel, showed that emergency medical personnel decisions to transfer patients to medical centers depend on the patient’s condition (physical, socio-economic and cultural health status), the characteristic of the mission (time, equipment and access to counsel) and personal characteristic (ability to reason, physical health, and collateral supporting) [16]. The results of the study on the response of paramedics when faced with cardiac arrest terminal patients showed that, when paramedics have freedom to choose whether or not to start CPR, they are more likely to respect the wishes of patients and families regarding the DNR (do not resuscitate) [17]. To make decisions when faced with ethical challenges, pre-hospital personnel use different approaches for moral reasoning [6], varying support resources (colleagues, regulatory authorities, codes of ethics or family members), and unique solutions [18]. The findings of a literature review have indicated that the moral reasoning and decision-making process of nurses and their ethical behavior are not a mere cognitive process [19]. Also, the decision making process is influenced by some other personal issues (personal values, experiences, knowledge and skills) and contextual values (expectations and comments of other nurses, family or doctor) [19]. In addition, in their ethical reflection, nurses are inspired by their emotions, intuition, and personal experiences. They sometimes consult with their colleagues when faced with moral dilemmas, and sometimes have separate ideas [20]. The results of some research suggest that the response of some pre-hospital personnel in dealing with ethical challenges is affected by various factors such as paying attention to the values and wishes of the patient, professional and organizational values, socioeconomic factors and cultural factors, as well as the boundaries of the therapeutic relationship with the health team and patient [7, 21, 22]. Given that the qualitative research is aimed at describing the understanding of human experiences and achieving a deep insight by improving the human perception and its exploratory nature in describing a phenomenon [23], it appears that qualitative studies are more effective than quantitative research in the study of the individuals’ responses to challenging situations. On the other hand, since a few number of studies have been conducted in Iran on the experiences of EMS personnel in the field of EDM, the present study aimed to identify and describe the experiences of EMS personnel in EDM when they are faced with ethical dilemmas. In addition, this study aimed to identify and describe the experiences of Iranian pre-hospital emergency service personnel in the field of EDM.

## Methods

### Study design

This qualitative study was performed in Iran, using the content analysis approach. The knowledge generated using this method is based on a unique perspective of participants and actual data in text. In other words, in this method, codes and categories are directly and inductively obtained from raw data [24].

The conversations took place at the ambulance departments affiliated with the University of Medical Sciences in late spring 2015. After telephone contact and initial coordination with the participants, 14 EMS personnel were identified by purposive sampling and invited to participate in this study. The inclusion criteria for participation in the study included at least 3 years professional experience in pre-hospital medical emergency centers.

### Data collection

In-depth semi-structured interviews were used for data collection. All the interviews were conducted individually in a private quiet room at the emergency medical department. Interviews were recorded on an MP3 player and later transcribed verbatim by the first author. The interviews started with broad questions at the beginning of the interviews, such as “could you tell me about the ethical dilemmas you encountered”. Then, in order to better understand and identify the EDM obstacles, other questions were raised in accordance with the objectives of the research. The questions included “What did you do when you were in that position?”, “What was your reason for making the decision?”; “can you tell me more about it?” In order to obtain a clear image of EDM in EMS personnel, all the interviews were conducted by the same researcher, and lasted between 45 to 70 min (mean = 63). Data collection ended when theoretical saturation of the data was achieved [25].

### Data analysis

Data analysis was conducted concurrently with their collection, using the 5-stage method proposed by Graneheim and Lundman and included the immediate transcription of the data after each interview. In order to reach a general understanding of participants’ statements and determine the content hidden in the data, recorded interviews were read several times with the aim in mind, with notes being made in the margins, in order to begin content structuring depend [26]. Meaning units were extracted from the text, condensed, and categorized at the same time based on their similarities and differences and through the constant comparison method. The concept and content hidden in the data were ultimately extracted and the final codes, classes and categories then emerged with the research team’s criticism, analysis and grouping of the codes.

### Rigor

The credibility of the study’s findings was established by varying the participants regarding age, gender, work experience, prolonged engagement with the participants, the member- and the peer-checking techniques. In addition, the results were given to 2 EMS personnel who did not participate in the study, and they were asked to compare the results with their work experiences. To perform peer check, the codes and themes obtained from the findings of this study were given to 2 faculty members specializing in qualitative researchers, and they approved the accuracy and conformity of the data with the codes and categories. Transferability was attained by detailed descriptions of the data and research process, which enabled the readers to judge accuracy and match the findings with their own contexts. Furthermore, regarding dependability in this study, 2 outsider observers were asked to survey and approve the data collection and analysis processes. The gleaned data were documented in all stages of the study as a detailed report to help confirm the research.

## Results

Participants of the study included 14 EMS personnel, with at least 3 years of work experience within an age range of 26–43 years. They included 3 emergency medical technicians (EMTs), 4 individuals with Bachelor of Science (BS) in EMS, 5 with BS in nursing, and 2 with Master of Science (MSc) in nursing. After data analysis, About 340 initial codes were extracted from all the interviews. The codes were summarized after undergoing review several times, and were classified because of similarity and proportion. Three main categories were extracted from the interviews: “respecting client’s values”, “performing tasks within the professional manner “, and “personal characteristic”. In the process of data analysis, 8 subcategories were obtained which, in turn, fell into three main categories (Table 1).

**Table 1:** Main categories and subcategories of experiences of EMS personnel in EDM

Main categories	Subcategories
Respecting client’s values	Protecting privacy/confidentiality
	Respecting client’s opinions and beliefs
	Respecting client’s wishes.
performing tasks within the professional manner	Professional commitment
	Maintaining the professional dignity
	Performing the tasks within the framework of the regulation
Personal characteristic	Inner values and beliefs
	Professional experiences

### Respecting client’s values

This main category was extracted from the three subcategories of “protecting privacy/confidentiality”, “respecting client’s opinions and beliefs” and “respecting client’s wishes”.

#### Protecting privacy/confidentiality

Everyone has privacy, a sense that every adult person has of his/her identity, dignity, independence, and personal space. In the field of medical science, especially in medical emergency services, one of the major issues faced is that of respecting the privacy of individuals both by physical contact and in privacy issues such as confidentiality, patient privacy and keeping his / her dignity. In the present study, the majority of issues which pre-hospital personnel were faced with included the patient’s nakedness at home or on the street; disrobing a part of the body for examination, therapeutic intervention or cardiopulmonary resuscitation, and respecting the patient’s privacy to private consultation and expressing his/her feelings.

*There was a case of gas inhalation injury and a woman's husband carried her nude out of the bathroom . . . we immediately requested for a blanket and took her to the hospital properly covered. Respecting patients’ privacy makes us to feel more relaxed and have a strong sense of work ethic. We suppose that this event could also happen to us. Participant no. 7 (EMTs)*

*Members of a family fell off a road. A woman without veil was breastfeeding her child and some people gathered around her. . . I immediately wrapped a blanket around her, took her into the ambulance, and assured her that she should not worry about anything and that she should just think of her baby. While doing this, a police officer wanted to enter the ambulance to get report but I did not allow him. Participant no. 5 (MSc in nursing)*

#### Respecting clients’ opinions and beliefs

The first step in promoting professional ethics and quality of care is by agreeing on opinions, values, and ethical norms from the patients’ perspective. Results have shown that EMS personnel try to understand the culture and customs of the area of missions (the place where personnel are called to provide emergency services) and adapt themselves with the customs of their client, before any intervention. Sometimes, EMS staff use the participative decision making with their client while performing transfer or treatment to express respect for the client’ values and beliefs.

*Sometimes in a family, besides providing the treatment, you should respect their values with your speech and behavior, since it may cause problems. They have some beliefs . . . they may challenge you (they may complain about you) although you are right; you need to have dual-energy to resolve the problem. Participant no. 12 (BS in nursing)*

*We were aware of their cultural and economic situation. For burial there, death certificate is not required; on the other hand, the transfer of dead bodies was not our task. However, since it was midnight and her family was confused, and in the culture of the region, keeping the corpse on the ground was not good, the corpse was transferred to the hospital* ***.*** *Participant no. 6 (BS in EMS)*

#### Respecting client’s wishes

From their missions, the participants’ experiences indicated that in their decision-making process, they respect the patients and their family’s desires and wishes as well as meet their needs; as long as it does not cause a deterioration in the patient’s health and it does not have a legal consequence for them.

*An elderly woman who had no particular problems called the emergency. We asked her where the patient was and what her problem was. She answered that there was no problem, it was just her birthday, and she was alone, hence she decided to call us to come and take part. We accepted her invitation and stayed with her after informing the emergency center. Participant no. 10 (BS in EMS)*

### Performing tasks within the professional manner

This main category was extracted from the three subcategories of “professional commitment”, “maintaining professional dignity”, and “performing tasks within the framework of the regulation”.

#### Professional commitment

Participants of the present study believed that this profession dealt with individuals’ life, property, and honor. Hence, they should respect the professional and human values as well as the altruistic services, since they have some authority in this regard. Feeling compassion towards a fellow and providing humans the services in all circumstances are among the principles that EMS personnel have attempted to consider in their decision-making process.

*Although it was not an emergency case and we could not transport the patient, but his family expected us to help them . . . It is out of humanity and value to give a negative answer to the request of a family who has suffered from pain for several years. Participant no. 3 (EMTs)*

EMS personnel try to be committed to their profession and maintain the professional values of their work, since they have accepted some responsibilities that need to be carefully performed.

*We are obliged to control the ambulance's equipment in terms of being complete and intact. Sometimes, when we are in an emergency case such as a car accident or CPR case, if we do not have enough medicine, then it will not be compensated. Participant no. 14 (BS in nursing)*

#### Maintaining the professional dignity

Professional dignity can be defined as a complex concept consisting of social elements and innate characteristic of the person. EMS personnel respect the professional dignity in care every day, especially in relation with pre-hospital care recipients and other professionals. The first aspect is related to the maintenance of patients’ dignity, which is presented as an inner value by EMS staff. The second aspect is related to the maintenance of the professional values and status. EMS personnel believe that respecting the values of patients, showing respect in inter- and intra-professional relationships, and performing professional roles appropriately can enhance their professional and social status. EMS personnel respect their professional dignity and show the importance of their presence in the care of those who are in need of their help, by observing appropriate coverage and appearance, having suitable behavior and good contact with patients and other members of the health care team.

*Because of this uniform that allow me to enter into the patients’ bedroom, even the police are not allowed to enter and touch the patient; without the uniform, they even don't let us to enter into their house. In some cases, we could not enter into the house of families easily because of some religious excuses, but if we behave seriously, they will not resist and will trust us. Participant no. 4 (EMTs)*

*In that scene, in spite of the great insults of the people around us, we tried to maintain our professional dignity and talk to them in full respect. Participant no. 5 (BS in nursing)*

#### Performing the tasks within the framework of the regulation

One criteria for the pre-hospital staff *in* performing the assigned duties and in EDM situations is to use the legal guidelines and circulars issued by the *disaster management and emergency medicine center*. The uncertainty of personnel in EDM situations and getting rid of the consequences of violating the law while performing the assigned tasks are among the reasons for the staff to make decisions within the framework of the regulation.

*. . . We immediately wanted to begin CPR; one of the relatives said that we should not bother him, if there is no hope for his survival. I asked about his relation to the patient and realized that he was legally ineligible to make a DNR order, hence we started the CPR. If we had listened to him, it is possible that the main legal guardian would have complained that we did not do the CPR. Participant no. 11 (EMTs)*

*The main factor which affects our work is the law because eventually, in any complains, the law will take its course. In any situation, I always consider the law, in order not to say anything that will put me against the law. Participant no. 8 (BS in EMS)*

### Personal characteristic

The third category composed of two subcategories as “inner values and beliefs” and “professional experiences”.

#### Inner values and beliefs

Some of the EMS personnel decide based on their beliefs, inner values, and conscience when dealing with ethical challenges. However, making decision based on conscience and inner values is associated with some challenges and may be in conflict with the values of the patient or organizational rules.

*In decision-making, first, I consider my conscience; whether the transfer of the patient to the hospital is true or not or if God is pleased with my job or not? Participant no. 13 (MSc in nursing)*

*In a car accident, when we arrived at the scene, there was a woman with inappropriate clothing. At first, I put a blanket on her, because my conscience told me to cover the woman in order to make her feel comfortable. Participant no. 6 (BS in EMS)*

In ethical situations, the pre-hospital personnel often put themselves in the shoes of client and try to do what they like others to do to them in the same situation.

*I did not get into the room and ask someone to put something on the patient, it is true that we are considered as confidants, but in any case, I don’t feel comfortable; this problem is not for others only but it might also happen to us one day*. *Participant no. 14 (BS in nursing)*

#### Professional experiences

One of the factors that play a pivotal role in EDM process of the emergency personnel is the knowledge and experience that they gained in the face of ethical challenges. According to the participants, the greatest factors that play a facilitating role in making ethical decisions were related to personal experiences gained in the missions, working with expert colleagues, and experiences gained from other colleagues*.*

*I received a complaint from a patient's family, when I was a novice and I did not know much about their culture and beliefs. Nevertheless, later on, I encountered such cases several times and my experiences increased, now, I do not have any problem dealing with the sensitivities and beliefs of patients. Participant no. 4 (EMTs)*

*In that situation, I lost my concentration and my expert colleague spoke intelligently with one of the relatives of the patient and decided to transfer the patient. Participant no. 5 (BS in nursing)*

## Discussion

Results obtained from analysis of the statements on the participants’ experiences showed that EMS personnel have a special framework in performing pre-hospital care, especially when faced with ethical challenges. In the present study, one of the main indicators for providing pre-hospital care was respecting the values of the client including sub-categories of “protecting privacy/confidentiality”, respecting client’s opinions and beliefs, and their wishes”. Respecting patient’s privacy can be viewed from two aspects: first, respecting the patient’s privacy by the ambulance technicians or nurses themselves, and second, maintaining the patient’s privacy in the public and other people at the scene. In their missions, when entering patients’ houses, EMS personnel encounter various scenes and their reactions are based on the previous knowledge and experience, moral sensitivity, individual moral philosophy, values and belief. Sometimes, they try to put themselves in the client’ position and meet their expectations as they like to be dealt with in the same situation. The clients, who are under the care of the treatment team, have rights including the respect for privacy in all stages of care. One’s privacy is a feeling that he/she has about his/her identity, dignity, independence, and personal territory [27], and it determines the transfer rate of an individual’s thoughts and feelings to others [28]. Yura et al. attached great importance to privacy of a patient and they believed that this issue helps to develop personal independence and identity, and human growth [29]. In addition, respecting personal privacy is necessary for creation of effective communication with the patient and maintaining their calmness and satisfaction level [30]. Since in pre-hospital missions, it is not possible to perform the care plan for individual with the same gender, EMS personnel are faced with more challenges and sensitivities in respecting the privacy of the patients. Cases such as accompanying the patient at his/her bedside at home or in the back cabin of the ambulance, lack of personnel of both sexes, low awareness of the rights of the client, priority to save the patient’s life, the relatives’ sensitivity and psychological stress caused by their presence on the scene is probably involved in failing to protect patient’s privacy. The results of the present study showed that despite the limitations, the participants tried to maintain the privacy of patients, unless saving patients’ lives is a priority. It can be rooted in the culture and religious beliefs of participants. In some resources, respecting client’s privacy has been presented as a fundamental component of a holistic care in meeting the needs of an individual; this can give the patient dignity and create a range of mutual trust [31]. The results of the study conducted by Raee et al. showed that factors such as shortage of manpower, casual look at the needs of patients, and lack of physical space are among the barriers to protecting the patient’s privacy by nurses [32]. The results of some studies indicate the invasion of privacy of patients and violation of the territory of the patient private information in the emergency department [33]. The different results on violation of patient’s privacy can be related to underlying conditions that the medical team personnel are faced with. Respect for patients’ privacy and their families by Iranian EMS personnel can be rooted in faith and religious beliefs. Most of them, in their decisions, consider God’s satisfaction.

The EMS personnel try to perform their task while respecting the wishes of the patients and their beliefs, because they believe that respecting client’ wishes increases their cooperation level in the process of treatment and reduces the negative consequences. In the qualitative research carried out in Iran by Ebrahimian et al. to analyze factors affecting the transfer of patients by EMS personnel, it was reported that some characteristics in this regard include the cultural background, beliefs, and attitudes of patients and their families [16]. Although, Iranian nurses respect patients’ beliefs and values [34], the results of several studies have shown that 5–10% of paramedics are forced to perform unwanted CPR when faced with cardiac arrest [35]. These conflicting results in accepting the client and relatives’ request can be related to the condition of the patients, the legal consequences of the decision and the atmosphere of the scene.

Performing the tasks within the professional manner is also another major category obtained from the findings, which includes sub-categories of “professional commitment”, “maintaining the professional dignity” and “performing tasks within the framework of regulation”. In their contacts with patients, EMS personnel face different ethical challenges such as client’ high level of expectations and demand (transferring the non-emergency cases to hospital, refusal of treatment), presence of relatives, standards of the profession and organizational structures (protocols) [5]. In the present study, these challenges were also seen in the EDM process by the EMS personnel; they respect the patient’s values and their professional dignity follows the regulation framework. However, sometimes, these values are in conflict with each other. Iranian nurses have mixed the nursing care with some ethical concepts like sympathy, compassion, commitment to the patient’s health, and self-sacrifice [36]; serving people and showing empathy and affection to the patients are allowed if it does not hinder the treatment process [37].

According to the findings of this study, EMS personnel are committed to performing their professional duties. In addition, they respect their professional dignity and perform their duties in a framework of rules. The professional commitment shows the individual’s loyalty to the profession [38] and committed individuals follow the professional goals and values and are proud of their profession; they consider their career as an integral part of their lives. In fact, the organizational commitment is an emotional dependence on the organization, that is to say, committed employees take their identity from the organization, and they are involved in the organization and enjoy their membership in the organization [39]. In the present study, the participants considered themselves part of the organization and were committed to their duties. Jafaraghaei et al. in examining the experiences of nurses in professional commitment found that many nurses consider helping others as a basis for creating professional commitment and effort for that, especially in the most critical conditions. They believe that there is no such condition in any other profession for individuals; this attitude is largely rooted in the nature of Iranian society. Iranian society is linked with spirituality and religious beliefs and some nurses consider their profession as a divine destiny for themselves which make them to be more spiritual [40]. The results of a qualitative study showed that clinical nurses in Iran consider their commitment to be a result of their spiritual beliefs [41]. According to this study, performing the tasks in the framework of the regulation has less consequence on technicians and in this case, they can defend themselves by referring to the law. However, referring to the law or considering the ethical values in decision-making depends on the scene conditions. For reasons such as the pressure of relatives, maintaining their safety, and lack of system’s support, emergency personnel sometimes had to transfer the patients and make some interventions which are outside the scope of their responsibilities. For instance, because of the requests of the patient’s family at the scene, some EMS personnel do some interventions and CPR despite the signs of imminent death or in cases where the family knows that the patient does not need the CPR [42]. Hence, strict adherence to the law can sometimes conflict with the requests of patients, families, and even inner feelings of the personnel. To prevent the consequences of their decisions, nurses sometimes prefer to stick to the law than to listen to their inner voice [43]. Ebrahimian et al. in their study showed that the support system of EMS personnel including the law, profession, financial support, and insurance coverage are among the factors influencing their decision-making process at the scene [16]. Therefore, commitment to the profession and maintaining professional values are ​​affected by various factors that are associated with the system supports, legal consequences of a decision, or individual characteristic which will be mentioned in the next category.

Another major category that is influential in the EDM process by the pre-hospital personnel is personal characteristic. Decisions made by the EMS personnel can be influenced by their inner values and beliefs, knowledge, and their professional individual experiences. Personal values are a set of beliefs that individuals hold in their world consciously or unconsciously and it covers some ideas that make them to prefer a particular form of behavior to other behavioral options [44]. In fact, personal values are the concepts or beliefs on the desirable goals or behaviors of individuals that guide them in a given situation to select or evaluate the behavior of others [45]. In the present study, some personnel mentioned that their adherence to the inner conscience and beliefs was involved in their decision-making process and this issue can be rooted in the cultural and religious beliefs governing the Iranian society. Findings of the study conducted by Ebrahimi et al. showed that some characteristics such as belief in doing the right thing, loyalty, sense of altruism, and moral sensitivity could affect ethical behavior of nurses far beyond their legal obligations during the decision-making process [46]. Based on the findings of Goethals et al., values and beliefs of nurses influence their moral behavior more than the rules and organizational expectations [19]. The results of these studies are not fully consistent with the findings of the present study. This may be due to the different background and contextual conditions of the participants; in general, they prefer the moral values to the organizational rules. Professional experience is one of the sub-categories of individual characteristic that play a pivotal role in the process of EDM based on the findings, the experiences gained from previous missions or expert colleagues play the most important role in dealing with ethical challenges. Expert individuals have various interpretations of situations and they make more appropriate decisions in critical situations. Expert EMS personnel have more intelligent response in dealing with sensitive situations. Many of the health professionals make decisions based on their internal and moral arguments when faced with ethical challenges. Borhani et al. in their study indicated that there is a positive relationship between the work experience and ethical reasoning of the nurses [47]. Furthermore, the findings of Ebrahimian et al. showed that one of the factors that affect EMS personnel’s decision-making at the scene is characteristic of personnel that include three main concepts: reasoning ability, support systems, and physical conditions. They also suggested that personnel with higher level of knowledge and experience have greater ability to make good decisions in critical situations [16].

## Conclusion

The results of this study revealed that EMS personnel are highly sensitive to protection of client’ privacy and respecting their beliefs and wishes; it can be rooted in culture, faith and religious beliefs of the Iranian participants. Pre-hospital providers put themselves in the shoes of client and try to do what they like others to do to them in the same situation. In their decision-making, they usually consider professional values and principles, and organizational rules; also, they are committed to assigned tasks. Performing the tasks within the regulation framework by EMS staff can be related to possible negative consequences of non-observance of the law. Pre-hospital providers consider the legal consequences before making any decision; because they usually make decisions in uncertainty conditions, in which the authorities’ support is inadequate and there is no specified protocol in complex situations. Hence, pre-hospital providers act more cautiously and examine possible consequences of their decisions. This study also revealed that previous experiences of pre-hospital personnel play a pivotal role in EDM. Furthermore, the participants pointed out the importance of working with expert colleagues. This study showed the various aspects of EDM of EMS personnel in the Iranian society. Authority’s awareness of these aspects may be helpful in improving EDM process in EMS staff.

Although, the participants shared their experiences with the researchers, this study has some limitations. This study was conducted in a limited number of pre-hospital emergency centers, each of the centers were different from each other in terms of medical equipment, staff’s work experience, mission characteristic and the culture of the people who live in these areas. Hence, the findings cannot be generalized to other centers of EMS and the findings are not applicable to all communities. Therefore, it is suggested that such qualitative research be conducted in other pre-hospital centers in Iran.

## Abbreviations

CPR: Cardiopulmonary resuscitation
DNR: Do not resuscitate
EMS: Emergency Medical Services
EMT: Emergency medical technician
PEC: Pre-hospital emergency caregivers
PHEMS: Pre-hospital emergency medical services
